# Electrophysiological Measurements of Peripheral Vestibular Function—A Review of Electrovestibulography

**DOI:** 10.3389/fnsys.2017.00034

**Published:** 2017-05-31

**Authors:** Daniel J. Brown, Christopher J. Pastras, Ian S. Curthoys

**Affiliations:** ^1^Neurotology Laboratory, Sydney Medical School, The University of SydneySydney, NSW, Australia; ^2^Department of Psychology, The University of SydneySydney, NSW, Australia

**Keywords:** vestibular, VSEP, electrovestibulography, electrocochleography, microphonic

## Abstract

Electrocochleography (EcochG), incorporating the Cochlear Microphonic (CM), the Summating Potential (SP), and the cochlear Compound Action Potential (CAP), has been used to study cochlear function in humans and experimental animals since the 1930s, providing a simple objective tool to assess both hair cell (HC) and nerve sensitivity. The vestibular equivalent of ECochG, termed here *Electrovestibulography* (EVestG), incorporates responses of the vestibular HCs and nerve. Few research groups have utilized EVestG to study vestibular function. Arguably, this is because stimulating the cochlea in isolation with sound is a trivial matter, whereas stimulating the vestibular system in isolation requires significantly more technical effort. That is, the vestibular system is sensitive to both high-level sound and bone-conducted vibrations, but so is the cochlea, and gross electrical responses of the inner ear to such stimuli can be difficult to interpret. Fortunately, several simple techniques can be employed to isolate vestibular electrical responses. Here, we review the literature underpinning gross vestibular nerve and HC responses, and we discuss the nomenclature used in this field. We also discuss techniques for recording EVestG in experimental animals and humans and highlight how EVestG is furthering our understanding of the vestibular system.

## Electrovestibulography Background

The history of Electrocochleography (ECochG) as a technique for recording cochlear field potentials is well established (Eggermont, 2017), beginning with Wever and Bray’s (1930) recordings of the Cochlear Microphonic (CM) in response to air conducted sound (ACS) stimuli in cats, and the 8th nerve compound action potential (CAP) response shortly after by Fromm et al. (1935). Predominantly, ECochG is used to objectively monitor cochlear sensitivity to ACS in animal experiments. During the 1970s, ECochG evolved as a clinical tool for diagnosing 8th nerve schwannomas, for monitoring 8th nerve function during surgery, and for diagnosing endolymphatic hydrops, where the ratio of the Summating Potential (SP) to CAP ratio was of primary interest (Gibson et al., 1977). More recently, variants of ECochG have been used to monitor 8th nerve and hair cell (HC) function during cochlear implantation using the electrically evoked CAP (Scott et al., 2016), or have used the acoustically evoked auditory nerve neurophonic (Lichtenhan et al., 2014; Koka et al., 2017; Rampp et al., 2017) or the CM (Campbell et al., 2016) during surgery. It should be made clear that ECochG is not the name of a response *per se* (the response is the CM, CAP, ANN or SP), but rather the *process* of monitoring electrical potentials from excitable cochlear cells. Today, there is a decreasing reliance of ECochG in the clinical setting (Hornibrook et al., 2016), with the Auditory Brainstem Response (ABR; and variants of) and otoacoustic emissions primarily being used to objectively monitor patient hearing and an increasing reliance on diagnostic imaging.

Whilst ECochG is an established tool in hearing research, there is less appreciation for the vestibular analog of ECochG, which has been infrequently termed Electrovestibulography (EVestG; Charlet de Sauvage et al., 1990; Lithgow, 2012). EVestG may be considered the process of measuring electrical responses of the peripheral vestibular system. Analogous to the CM and CAP or ABR in ECochG, EVestG responses consist of both vestibular HC and vestibular nerve field potentials. Fluctuations in the extracellular potential due to movement induced changes in the vestibular HC conductance and receptor current has been termed the “Vestibular Microphonic” (VM), whereas the vestibular afferent nerve response (or central vestibular neuron response) to movement has been termed the short-latency Vestibular Evoked Potential (VsEP). This review article will focus on the VM and VsEP, as fundamental EVestG components.

EVestG has not been extensively used by inner ear researchers. That is, although the VM and the VsEP have been characterized, they are used far less often and rarely compared to their cochlear counterparts. A simple PubMed search for “vestibular VsEP” returns a list of just 49 publications, whereas a search for “cochlear CAP” or “cochlear CM” returns a list of 570 and 930 publications respectively^1^. Moreover, Electrocochleography is an established term, with more than 4000 publications listed on Pubmed, whereas the term Electrovestibulography has only been used in 20 publications, 18 of which were from the same research group. Some of this discrepancy may be due to variation in the nomenclature of these responses.

Over the last 20 years, the term Electrovestibulography has only been used to describe a recent controversial response that forms part of a patented recording technique (Lithgow, 2006, 2012). Here, Lithgow (2006) claim that the stochastically occurring field potential of the vestibular nerve can be extracted from the biological noise measured from the ear canal (i.e., this is not a stimulus evoked response *per se*). The authors use a signal analysis process to localize any stochastically occurring field potentials that have characteristics resembling the VsEP, occurring within the raw electrical recording from the ear canal. They then average these asynchronous field potentials, somewhat similar to the methods involving spike-triggered averaging (Kiang et al., 1976). To obtain a response that is dominated by vestibular activity, they accelerate the subject in a given direction for approximately 1 s. By subtracting the averaged field potential recorded during movement, from that without movement, the resulting difference waveform theoretically resembles a response of stimulated vestibular neurones. At present, there is only weak evidence to support the claim that such a response faithfully represents the activity of vestibular neurones, and other clinical or experimental researchers have not adopted the technique. Furthermore, the technique requires a complex system capable of performing a controlled acceleration of a person many times, synchronized with the recording condition. Fortunately, researchers have demonstrated much simpler techniques for objectively measuring peripheral vestibular function, via the VM and VsEP. Most of these studies have been performed in experimental animals, with a limited number of human studies.

## Response Nomenclature

Prior to reviewing how EVestG and ECochG measurements compare, there is perhaps a need to revisit, or clarify some of the terminology used in this field. Inner ear evoked responses, and more broadly electrophysiological responses, are rife with inappropriate nomenclature, although it would be impractical to alter their use today because they have been used for several decades. Nevertheless, it is necessary to have a clear understanding of how the electrical activity of excitable cells relate to extracellular potentials (Bressler, 2011; Buzsáki et al., 2012). A brief description of the major cochleovestibular electrophysiological responses, and stimulus “typically” used to evoke them is listed in Table 1.

**Table 1:** **Common cochlear and vestibular electrophysiological activity used to objectively measure inner ear function**.

Response	Stimulus	Latency (ms)	Source	Origin
Unitary potential	Spont.	N/A	Neuron(s)	The spontaneous field potential of a single neuron, or collection of neurons, measured distant to the cell. Requires special recording techniques to extract it from noise.
Neural noise or neurophonic	Spont. or ACS	N/A	Nerve	The ensemble electrical activity related to stochastic or cyclic activity of the 8th nerve.
**Compound action potential (CAP)**	**ACS**	**~1**	**Nerve**	**The compound summation of synchronously occurring unitary potentials**.
**Cochlear microphonic (CM)**	**ACS**	**<0.1**	**Hair cells**	**The field potential generated by hair cells. Typically recorded from the cochlear fluids**.
**Summating potential (SP)**	**ACS**	**<0.1**	**Hair cells**	**The charge imbalance (i.e., asymmetry) of the hair cell field potential, which is obtained by removing the symmetric components of the CM (either by stimulus inversion and averaging, or low-pass filtering)**.
Auditory brainstem response (ABR)	ACS	1–7	Nerve/ Brainstem	The compound summation of synchronously occurring neural activity in the auditory brainstem.
eCAP	Current	0–0.5	Nerve	An electrically evoked CAP
Middle and long latency response	ACS	10–500	Cortex	The compound summation of synchronously occurring neural activity in the auditory cortex.
Post-auricular muscle response	ACS	12–20	Myocytes	A compound summation of the electrical response of the post-auricular muscle.
Frequency following response	ACS	N/A	Nerve/ Brainstem	The ensemble electrical activity related to cyclic activity of the auditory brainstem.
**Vestibular short latency evoked potential (VsEP)**	**BCV**	**0.5**	**Nerve/ Brainstem**	**The compound summation of synchronously occurring neural activity of the vestibular nerve and brainstem**.
**Vestibular microphonic (VM)**	**BCV**	**<0.1**	**Hair cell**	**The field potential generated by hair cells. Typically recorded from the vestibule fluids**.
Vestibular evoked myogenic potential (VEMP)	BCV	10–20	Myocytes	A compound summation of the electrical activity of the extra-ocular or sternocleidomastoid muscles.

*Also provided is the typical stimulus for each response (Spont., Spontaneous; ACS, Air Conducted Sound; BCV, Bone Conducted Vibration, N/A, not applicable), and a brief explanation of the origin of each activity. Highlighted responses refer to those typically forming parts of ECochG and EVestG responses. The latency refers to the time after the onset of the stimulus, where the stimulus is evoked by the onset of a stimulus*.

These responses are all field potentials, generated by a subset of cells, evoked by a given ACS or bone conducted vibration (BCV) stimulus, whose response waveform differs with recording location and stimulus protocol. Unfortunately, most ACS or BCV stimuli will evoke a response from multiple cell-types (e.g., cochlear or vestibular neurons or HCs). For example, the CAP and VsEP can both be measured with electrodes in or near the inner ear, evoked by a BCV stimulus. Therefore, researchers might employ a technique, such as using moderate level transient ACS stimuli, with a low stimulation rate (e.g., 11/s), to maximize the contribution of the cochlear nerve to the field potential, and we may call this technique ECochG. EVestG is the technique of recording field potentials that predominantly reflect vestibular nerve or vestibular HC activity. Specifically, EVestG responses include the VM and the VsEP.

However, even the VM and VsEP may contain responses from different cell types. As discussed later, the VM may originate from either semicircular canal (SCC), utricular, or saccular HCs, and the VsEP may either reflect the compound activity of the 8th nerve, or central vestibular activity. It could be argued, for the purpose of consistency and to avoid confusion, that the VM should ideally be separated into SCC microphonic, utricular microphonic, or saccular microphonic, and that the VsEP recorded from the periphery should be re-termed the vestibular nerve CAP (as opposed to the cochlear nerve CAP), and that the VsEP recorded from the scalp should be re-termed the vestibular brainstem response. However, within this review we will continue to use the commonly accepted more general terminology, explicitly defining the recording location and origin of the response where appropriate.

## The VM and VsEP

Arguably, the greatest obstacle with performing EVestG measures and using them as a faithful measure of peripheral vestibular function is that both ACS and BCV stimuli can evoke cochlear field potentials (i.e., CM and CAP), which are an order of magnitude larger than vestibular responses, and will summate with the VsEP or VM. Selectively destroying the cochlea, which does not abolish the VsEP or VM, or destroying the vestibule, which does abolish them, provides clear evidence that these responses originate from vestibular sources. Researchers wishing to use EVestG without destroying the inner ear either need to suppress cochlear responses, or record responses at a location where cochlear activity is not present, or use a stimulus that does not stimulate the cochlea. There are a number of technical considerations when measuring EVestG responses, and a clear understanding of recording techniques is necessary when using EVestG as an objective measure of peripheral (or central) vestibular function.

### EVestG BCV Stimuli

Some form of transient or cyclic translation or rotation of the skull is commonly used to evoke the VsEP and VM. Often, this stimulus is transmitted to the head via an electromagnetic transducer or “modal shaker”, rigidly attached to the head. Whether the stimulus is a pulsed, cyclic, or angular translation of the head, here we consider all forms of head movement to be BCV stimuli. Other forms of vestibular stimulation include ACS, manual force applied to the head, or even force directly applied to the HC stereocilia, although this last method requires surgical exposure of the inner ear.

For the purposes of reproducibility and interpretation, it is necessary to measure the stimulus delivered to the vestibular system. Ideally, researchers could measure the movement of the vestibular end-organ directly (as has been performed in cochlear mechanics studies; Sellick et al., 1982; Chen et al., 2007), however this is impractical in most scenarios because the vestibular system is housed deep inside the inner ear. The next best, albeit indirect, option is to measure the movement of the skull, which can be achieved by rigidly attaching an accelerometer to the bone, skin, or to the modal shaker directly. However, with these indirect methods, the property of vibration through the skull needs to be considered.

The mechanical properties of BCV are complex, because the skull consists of rigid and compliable bone, combined with soft tissue and fluids. Additionally, the skull is segmented and separated by sutures, and has complex resonance features (Håkansson et al., 1994). Various attempts have been made to model and measure the properties of vibration transmission through the head, primarily in humans, and primarily aimed at understanding BCV hearing (Stenfelt, 2015, 2016). For the human head at least, the skull *approximately* moves as a rigid structure for BCV below 400 Hz (Stenfelt and Goode, 2005), as a resonant structure between 400 Hz to 2 kHz (Håkansson et al., 1994), and as a wave-propagating structure above 2 kHz (Stenfelt, 2015). These parameters solely relate to the propagation of vibration through the bone, and do not include the additional compliance of soft tissues like skin, or the fluid dynamics of the inner ear known to play a role in HCs stimulation (Sohmer et al., 2000; Sohmer and Freeman, 2004; Stenfelt, 2015). Moreover, there is little information regarding BCV through experimental animal heads, which will have vastly different mechanical properties to that of human skulls. Ultimately, it should be made clear that, particularly for pulsed or cyclic (>100 Hz) BCV in experimental animals, that movements measured on or near the skull are unlikely to faithfully represent the vibration of the vestibular HCs. Moreover, particularly for high-frequency (>400 Hz) BCV, the head movement is likely to differ when measured at different locations (Durrant and Hyre, 1993). Without a standard BCV measurement technique, it can be difficult to compare head movements between studies. Thus, whilst researchers can directly measure otolith sensitivity to different BCV frequencies, caution should be taken when interpreting the response properties of the end-organ itself, particularly when the BCV stimulus is delivered to the head at different locations and under different conditions.

At one level, ACS stimulation of the vestibular system may be easier to interpret, because the bulk of the energy is transmitted through the ear canal where sound levels can be measured as a standard, and a great deal of work has been done on ACS transmission through the middle-ear (Ravicz et al., 2010). The frequency response of ACS stimulation of the otolith neurons closely resembles middle-ear transmission frequency response, although there are differences in the sensitivity of the different vestibular end-organs. How ACS stimulates the vestibular system is less clear, although it presumably involves fluid pressure waves inducing displacements of the vestibular HCs or their stereocilia. The problem with ACS stimulation for EVestG measurements however, is that cochlear HCs are 100 dB more sensitive to ACS than vestibular HCs, and relatively large ECochG responses will be present in ACS evoked field potential recordings.

### VM Recordings

The VM was first reported just 8 years after the CM in 1938, albeit in an *ex vivo* preparation (Adrian et al., 1938; Zotterman, 1943; Lowenstein and Roberts, 1951; Wever and Vernon, 1956). Since then, the VM has been recorded *in vivo* in zebrafish (Trapani and Nicolson, 2010; Yao et al., 2016), toadfish (Rabbitt et al., 1995), bullfrogs (Eatock et al., 1987), pigeons (De Vries and Vrolijk, 1953; Wit et al., 1986, 1990), and guinea pigs (Trincker and Partsch, 1959). The VM reflects changes in the receptor current through the mechano-electrical transduction channels located on the stereocilia of the vestibular HCs, which are displaced due to inertial drag, resulting from a shearing force that displaces the otoconia or cupula (Fernández and Goldberg, 1976).

#### *Ex Vivo* VM

**Figure 1:**
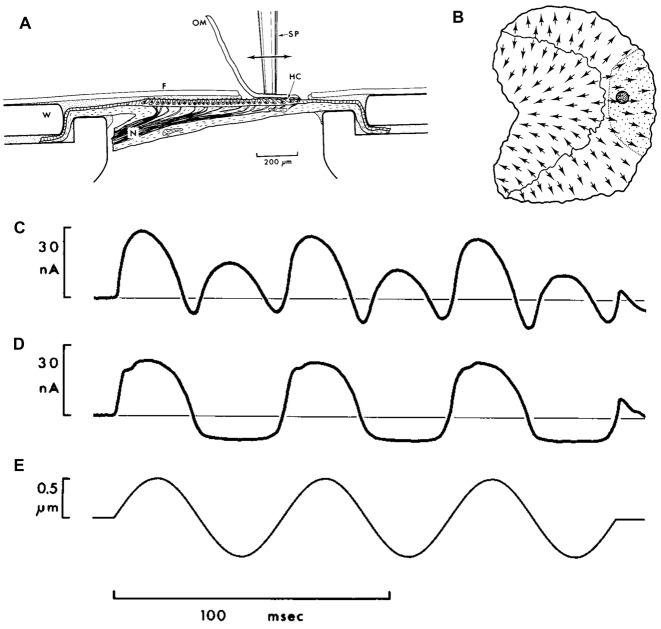
**(A)** *Ex vivo* Vestibular Microphonic (VM) recordings from a bullfrog’s saccular maccula. The macula has been extracted and placed between two fluid-filled baths, sealed with a washer (W) in an Ussing-chamber format. A thin plastic film (F) isolates a region of the macula exposed to the bath. The fluid potential between the baths is used to provide a measure of the VM. The macula is vibrated via a stimulating probe (SP) directly attached to the otolithic membrane (OM), which is either intact, or partially removed from the macula such that it only adheres to hair cells (HCs) of a single orientation. **(B)** A schematic illustrating of the saccular macula, with arrows indicating HCs polarities, and highlighting the location of the probe (dark shaded circle) and the area where the OM remains intact (shaded region on right of macula). **(C)** The VM response with the OM covering all HCs, demonstrating a response with twice the frequency of the vibration stimulus. **(D)** The saturated VM response, with the OM peeled back so that only HCs of a single orientation were stimulated. **(E)** The 16.5 Hz vibration stimulus. Reproduced with permission from Corey and Hudspeth (1983).

Much of our knowledge regarding the properties of HCs comes from *ex vivo* recordings of the VM from bullfrog otolithic HCs (Corey and Hudspeth, 1983; Azimzadeh and Salvi, 2017). Here, the otolithic maccula (most studies have used the sacculus) is extracted and placed between perilymph/endolymph filled baths in an Ussing chamber (Figure 1A; from Corey and Hudspeth, 1983), with a region of the epithelia exposed to both baths. Vibration is directly applied to the macula, or overlying otolithic membrane (OM), via a stiff probe (Figures 1A,B). Recording the bath potential provides a global measure of the VM generated from the HCs exposed to both baths (i.e., a summed response of all HCs), or alternatively intracellular potentials can be recorded with glass microelectrodes. VM recordings have been made with either the OM intact (Figure 1C), partially removed so as to only stimulate HCs with stereocilia of a particular orientation (Figure 1D), or totally removed. Removing the OM uncouples hair bundle motions from neighboring HCs, and has substantial effects on their excitability and sensitivity (Benser et al., 1993; Dierkes et al., 2008; Fredrickson-Hemsing et al., 2012; Ó Maoiléidigh et al., 2012). With the otolith membrane intact and all HCs are stimulated, the global VM will exhibit a response with twice the frequency of the vibration stimulus (Figures 1C,E). This is because HCs of both polarities are stimulated (Flock, 1965; Corey and Hudspeth, 1983). When only HCs on one side of the line of polarity reversal (Li et al., 2008) are stimulated the VM is cyclic, following the vibration stimulus (Figures 1D,E), although it will saturate at high stimulus levels (Hudspeth and Corey, 1977; Corey and Hudspeth, 1983).

Several other studies have examined the microphonic from the SCC HCs using an *ex vivo* preparation (De Vries and Bleeker, 1949; Van Eyck, 1951a,b,c; Masetto et al., 1995; Botta et al., 1998; Rabbitt et al., 2005). Here, the polarity of mechanical sensitivity is the same for all hair bundle stereocilia, such that mechanical displacements of the cupula either increases the conductance of all SCC HCs, or decreases it. This results in an asymmetrically distorted microphonic, which can be recorded some distance from the cristae in the vestibular fluids (Botta et al., 1998).

#### *In Vivo* VM

Few studies over the last 50 years have recorded the VM *in vivo*. This is arguably because evoking the VM requires low-frequency (10–1000 Hz) stimulation, which induces hair bundle displacements (Huizinga and Van Der Meulen, 1951; Trincker and Partsch, 1959; Bleeker et al., 1980; Wit et al., 1981, 1990), yet this will evoke a CM that will dominate the inner ear fluid potentials. That is, compared to VM responses, the CM is large (1–2 millivolts in the perilymph, and several times larger in endolymph; Honrubia et al., 1973) because there is a large electrochemical driving potential for the receptor current through cochlear HCs of +150mV (involving a +90 mV electrogenic potential on the apical surface, and a transmembrane potential of −60 mV; Davis, 1965), whereas the driving potential for the receptor current through HCs in the SCCs, utricle or saccule is most likely to be closer to +65 mV due to a much lower endolymphatic potential (Schmidt, 1963; Ono and Tachibana, 1990; He et al., 1997). Additionally, the CM is large because the polarization of HCs stereocilia sensitivity, within a given region of the cochlea, are aligned in the same direction (Russell, 1983), and cochlea scalae are separated by an epithelium with an electrical impedance of 40–50 kOhm (Johnstone et al., 1966). Conversely, the otolith HCs microphonic will cancel in the fluids due to opposite polarity of HCs either side of the line of polarity reversal, which generates microphonic potentials in the fluids which are 180° out of phase (Corey and Hudspeth, 1983). Furthermore, vestibular HCs are either supported by bone-anchored epithelia, or in the case of the utricle, suspended on a membrane which most likely has an electrical impedance close to 13 kOhm, and therefore the circuit potential related to vestibular HC stimulation will be comparatively low.

Most *in vivo* studies of the VM have necessarily abolished cochlear function prior to monitoring the VM, and have measured the VM within the inner ear fluids (Adrian et al., 1938; Wever and Vernon, 1956; Trincker and Partsch, 1959; Wit et al., 1981, 1986, 1990). Only a few studies, mostly using fish, have recorded the VM without destroying the cochlea (Zotterman, 1943; Furukawa and Ishii, 1967; Fay and Popper, 1974; Rabbitt et al., 2005; Sisneros, 2007; Yao et al., 2016). VM recordings in fish, particularly zebrafish, are emerging as a powerful tool for studying inner ear developmental biology (Trapani and Nicolson, 2010; Yao et al., 2016). Here, both the lateral line organ and the inner ear (the otic capsule) will respond to alternating pressures and generate microphonic potentials, and differentiating the source of the VM (i.e., explicitly which HCs generate the VM), will be complex due to the small size of the organ.

**Figure 2:**
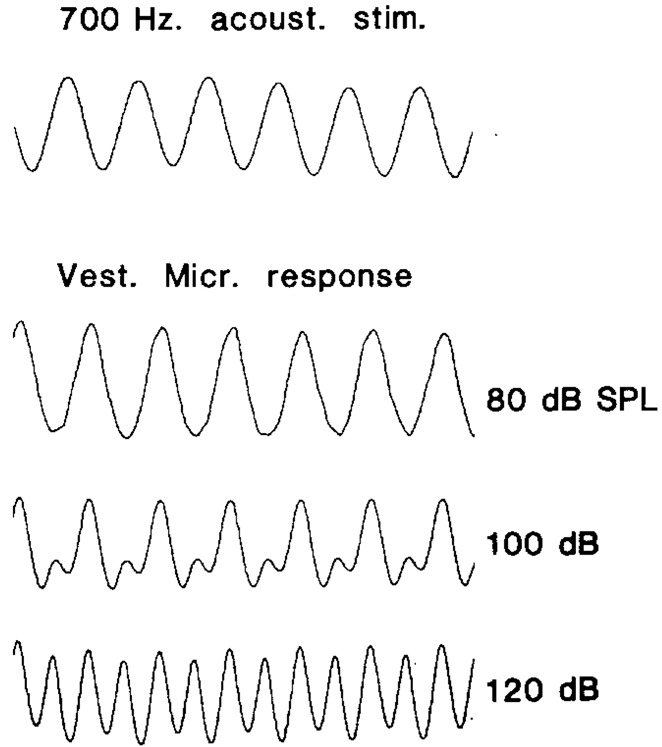
**Scala tympani recordings of the VM recordings in pigeons, in response to a 700 Hz air conducted sound (ACS) tone (upper trace) after cochlear extirpation and semicircular canal (SCC) fenestration**. At low sound levels, the VM (lower three traces) is a slightly distorted sinusoid, and as the stimulus level increases, so does the distortion, generating a response whose frequency is twice that of the stimulus. Reproduced with permission from Wit et al. (1986).

De Vries and Bleeker (1949) and Van Eyck (1949) were the first to measure VM *in vivo*, from the SCCs of pigeons. De Vries and Vrolijk (1953), used sinusoidal tympanic membrane displacements to evoke SCC microphonics in pigeons after the cochlea and otoliths had been destroyed. The otoliths were destroyed because they too were stimulated by displacement of the tympanic membrane, and the otolith responses contaminated the SCC responses. Here, the VM was recorded both in the vestibule, and in the SCC after a small hole had been made in the canal wall, which was shown to induce the Tullio effect and enhance SCC responses. Ultimately, the VM from the SCCs demonstrated phase relationships which supported Ewald’s laws, demonstrating highly nonlinear microphonic potentials, where each SCC was maximally stimulated for fluid motion in a given direction. Later Wit et al. (1986) used ACS stimuli, with a SCC fenestration and cochlear extirpation, to evoke VM responses in pigeons (Figure 2). Increasing the level of the stimulus resulted in the response frequency doubling, similar to that obtained with* ex vivo* experiments where the whole otolith was stimulated (Figure 1C), suggesting that additional vestibular HCs were being recruited with high level ACS, which had a response phase difference of 180°. No attempt was made to separate the response components.

Trincker and Partsch (1959) performed arguably the most extensive *in vivo* assessment of the VM in mammals, using guinea pigs, and stimulated microphonic potentials from the SCCs, utricle, and saccule, using both BCV and ACS tones, after the cochlea was completely destroyed. Recordings were performed with electrodes within the cochlear fluids, within the SCCs, or within the ampulla. Selective ablation of each end organ was used to confirm the specific origin of the microphonic. VM responses from all vestibular end organs were evoked with sinusoidal stimuli of frequencies between 300 Hz and 120 kHz. Given that CM responses are known to be evoked in mammals by sinusoidal stimuli up to 30 kHz (Cheatham et al., 2011), it seems highly unlikely that either cochlear or vestibular microphonic responses would have been evoked by the ultrasonic stimuli by Trincker and Partsch, and suggests that potentially some of the ultrasonic responses in their study may have included an artifact component.

Ultimately, whilst much research continues to utilize *ex vivo* measurements of vestibular HCs function, there is a need to substantiate the use of such *ex vivo* preparations as a reliable measure of the *in vivo* properties of vestibular HCs. Certainly for cochlear research, the CM remains a mainstay of experimental research measures, and has been used to support and further our understanding of the properties of HCs transduction, derived from intracellular receptor potential measurements (Patuzzi and Sellick, 1983; Patuzzi et al., 1989). For example, the *in vivo* CM has been used to demonstrate the underlying HCs related cause of many forms of sensorineural hearing loss (Patuzzi et al., 1989), which may have otherwise been attributed to neural dysfunction. Unfortunately, there has been little work done to establish techniques for measuring the VM *in vivo*, and most* in vivo* animal studies of the vestibular system are limited to measuring single-unit afferent responses (Fernández and Goldberg, 1976; Curthoys et al., 2006; Curthoys and Vulovic, 2011), single cell receptor potentials (Rabbitt et al., 2005), and VsEP responses (see below). Thus, our understanding of the origin of many forms of vestibular dysfunction may be lacking, as we have not utilized methods that may separate vestibular HCs from neural dysfunction. VM recordings offer an opportunity to perform simple recordings of vestibular HCs sensitivity *in vivo*, and may demonstrate changes that drive or differ from neural dysfunction.

### VsEP Recordings

The VsEP was arguably first demonstrated in 1949 in pigeons (De Vries and Bleeker, 1949). The VsEP has been further demonstrated in pigeon (Wit et al., 1981), chicken (Jones and Pedersen, 1989; Jones and Jones, 1996, 2000; Nazareth and Jones, 1998), canary (Jones S. M. et al., 1998), quail (Jones et al., 1997), mouse (Jones and Jones, 1999; Jones et al., 2006), rat (Lange, 1988; Plotnik et al., 1999a,b), chinchilla (Böhmer, 1995; Böhmer et al., 1995; Plotnik et al., 2005), guinea pig (Cazals et al., 1987; Jones and Jones, 1999; Oei et al., 2001; Kingma and Wit, 2010; Brown et al., 2013; Chihara et al., 2013; Bremer et al., 2014), rhesus monkey (Böhmer et al., 1983) cat (Elidan et al., 1987a,b; Böhmer, 1995), and human (Elidan et al., 1991a,b; Knox et al., 1993; Pyykkö et al., 1995; Rodionov et al., 1996; Loose et al., 2002). The VsEP has predominantly been evoked by a brief (2 ms) “linear” BCV pulse stimulus, with the response evoked by skull jerk rather than acceleration (Jones T. A. et al., 2011). It has mostly been recorded in experimental animals with a non-inverting electrode placed at the vertex, or within the facial nerve canal. The VsEP reflects the compound field potential of vestibular neurons (either peripheral or central), firing synchronously to the onset of a motion.

It is important to note that there are various VsEP recording procedures, and as a result, responses can reflect activity from different sources. Some recording protocols use linear-BCV pulses, whereas others use rapid head rotations. Moreover, the location of the recording electrodes significantly determines the VsEP waveform. The non-inverting VsEP recording electrode has been placed at various locations including the vertex (Elidan et al., 1982; Jones, 1992; Bremer et al., 2014), at different sub-cranial locations (Jones et al., 2002), within the vestibular nucleus (Cazals et al., 1987), within the facial nerve canal (Böhmer, 1995; Kingma and Wit, 2009; Bremer et al., 2012; Chihara et al., 2013), or on the round window (Aran et al., 1980). The inverting electrode is typically placed subcutaneously at a relatively non-responsive area such as the pinna or mastoid, and the ground (or common) electrode is placed at a distal location on the body, such as the neck. The characteristics of these different VsEPs, such as latency, waveform, and stimulus related phenomena also change with recording protocol. Importantly, all responses have short latencies (starting 1 ms to 2 ms) and remain after cochlear extirpation, but are abolished by damage of the vestibule or 8th nerve, or death (Jones and Jones, 1999). Moreover, the response is abolished via the application of neural blockers such as tetrodotoxin (Weisleder et al., 1990; Jones, 1992; Jones and Jones, 1999; Chihara et al., 2013), demonstrating that the VsEP is a neurogenic response. Any new VsEP recording protocol should first demonstrate that the response reflects the activity of the vestibular nerve.

#### Central vs. Peripheral VsEPs

**Figure 3:**
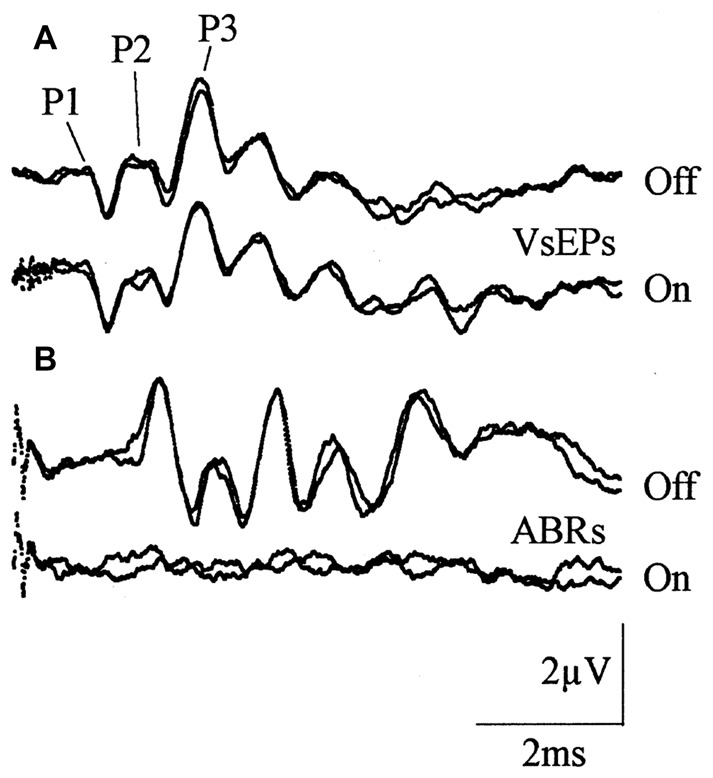
**Vestibular evoked potential (VsEP) responses recorded from sub-cranial vertex electrodes in mice. (A)** VsEP responses evoked by a 2 ms bone conducted vibration (BCV) jerk pulse, with and without forward acoustic masking, which does not alter the response. **(B)** Auditory brainstem response (ABR) responses with and without forward masking, demonstrating that ABR responses are forward masked. Reproduced with permission from Jones and Jones (1999).

The majority of VsEP studies have recorded the response with the non-inverting electrode placed subcutaneously at the vertex, or sub-cranially at different locations overlying the cortex. Here, responses typically start with a small (~0.5–1 μV) P1 peak (Figure 3A; which corresponds to the initial peak in facial nerve recordings; (Aran et al., 1980; Jones, 1992; Nazareth and Jones, 1998), and a series of slightly larger positive and negative peaks thereafter (Elidan et al., 1987a; Jones and Pedersen, 1989; Jones and Jones, 1999; Plotnik et al., 1999b; Bremer et al., 2014). This VsEP primarily reflects the response of various vestibular brainstem nuclei and nerves (Nazareth and Jones, 1998), much the same way the ABR reflects central auditory neuron responses (Figure 3B). Importantly, ACS evoked ABR responses are suppressed by acoustic forward-masking noise (Figure 3B), whereas BCV evoked VsEP responses are not (Figure 3A).

**Figure 4:**
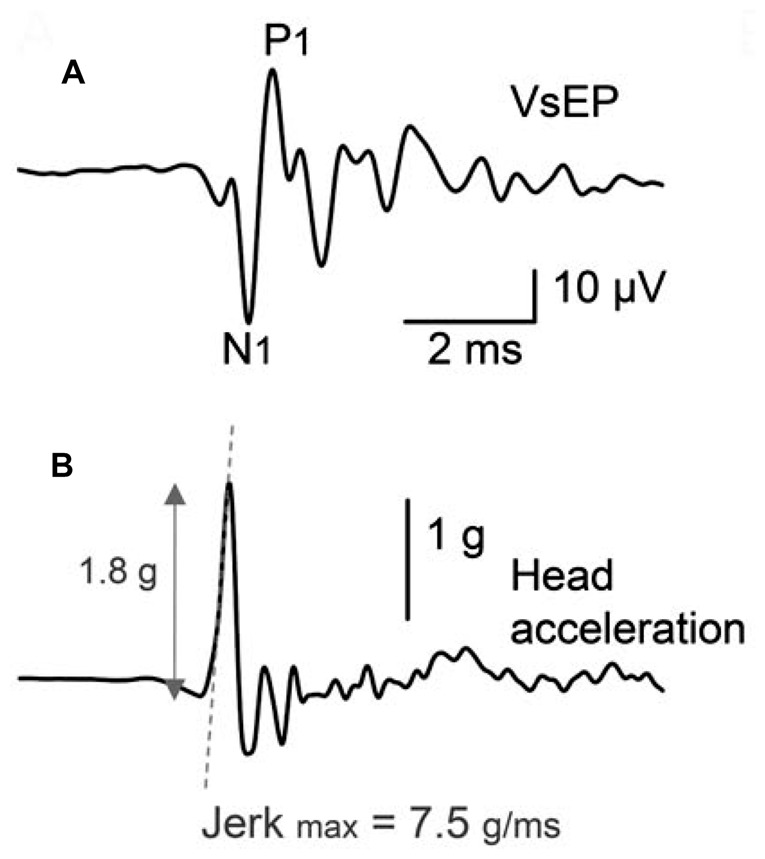
**(A)** Facial nerve canal recordings of the VsEP in an anesthetized guinea pig, in response to a brief, linear BCV click. Recordings were performed with the cochlea intact, and in the presence of continuous ACS masking noise. The VsEP consists of an initial negative peak (N1) and positive peak (P1), and a series of smaller peaks thereafter. **(B)** The acceleration of the skull, where the stimulus was designed to produce minimal oscillation of the head. Reproduced with permission from Chihara et al. (2013).

VsEP recordings performed with the non-inverting electrode within the cochlea or facial nerve canal will appear similar in waveshape to the cochlear CAP, with an initial negative and positive peak (with amplitudes between 20 μV and 100 μV), termed N1 and P1, with a few smaller peaks thereafter (Böhmer, 1995; Bremer et al., 2012; Chihara et al., 2013); Figure 4A). That said, other studies have suggested that VsEPs recorded within the facial nerve begin with a large positive peak (Oei et al., 2001; Kingma and Wit, 2009), and appear similar to an inverted version of a cochlear CAP. Regardless of the polarity of the first VsEP peak, this activity primarily reflects the compound field potential of the vestibular nerve.

#### VsEP Stimulus

**Figure 5:**
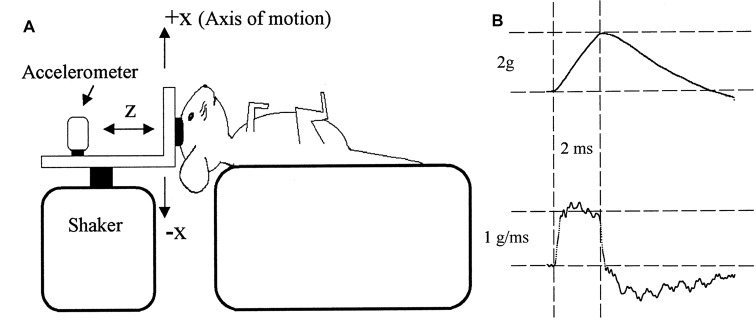
**(A)** The typical experimental setup used to evoke VsEP responses in animals. An electrodynamic modal shaker is attached to the animal’s skull via a screw or clamp. The acceleration is measured on the modal shaker connector. **(B)** Acceleration pulses rise gradually over 2 ms, producing a monophasic 2 ms jerk of the head. Reproduced with permission from Jones and Jones (1999).

The most widely utilized stimulus for evoking the VsEP involves delivering a rapid, linear-BCV impulse to the skull, in a naso-occipital direction, transduced by a large electrodynamic shaker bolted or clamped to the skull (Figure 5A). This theoretically permits a controlled, rapid push-pull of the animal’s entire head (with <100 μm displacement) in the naso-occipital direction. An extensive examination of the appropriate parameters for evoking the VsEP in mice and rats using this setup has been performed by Jones et al. (Jones and Jones, 1999; Jones et al., 2002; Jones T. A. et al., 2011). Here, it has been suggested that a rapid acceleration of the head, producing a 1 ms to 4 ms pulsed “jerk” (the derivative of acceleration; Figure 5B) is ideal for evoking the VsEP. Indeed, the level of BCV jerk, rather than the level of acceleration, velocity, or displacement, appears to be the main factor determining the amplitude of the VsEP response, and suggests the VsEP is a response of the primary afferents that innervate otolith jerk-sensitive HCs (Jones T. A. et al., 1998; Jones T. A. et al., 2011). Jones T. A. et al. (2011) also suggest that an ideal duration of the linear BCV jerk pulse is approximately 2 ms, which preferentially stimulates the vestibular system, with less cochlear activation. Most studies have demonstrated a reliable VsEP in response to a linear BCV stimulation between 0.5 g and 8 g, or 0.1 g/ms to 6 g/ms.

It should be noted that a 2 ms duration jerk pulse requires an acceleration pulse that increases from zero, peaks at 2 ms, and slowly declines thereafter (Figure 5B). The head velocity change will peak several milliseconds after the onset of the movement, and the peak displacement will occur several milliseconds after that (typically well after the VsEP has occurred). Such a movement of the head can be difficult to produce (particularly for larger heads), but may be necessary to maximally stimulate the jerk-sensitive HCs of the otoliths with minimal cochlear stimulation. Importantly, the head acceleration in this setup is measured on the mechanism attached to the shaker and skull, which arguably may not faithfully represent the acceleration of the vestibular system (Jones et al., 2015). That is, the otolith acceleration may be more complex than that recorded elsewhere in the system, given that the skull can compress and resonate in a complex manner in response to BCV pulses (Durrant and Hyre, 1993), and viscous forces act on the otolith organs (Jones et al., 2015). Moreover, it is not clear how much inter-aural or rostro-caudal movement of the skull is induced by a BCV pulse applied directly to the vertex in a naso-occipital direction.

**Figure 6:**
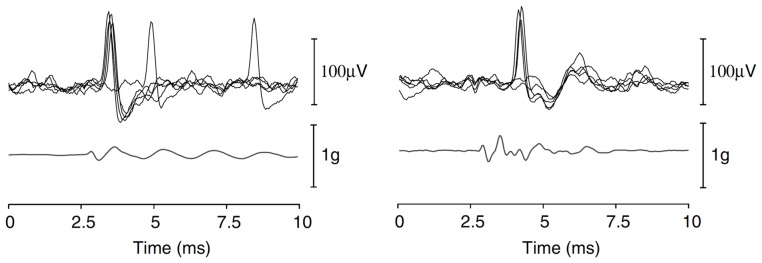
**Repeated single-unit responses from two pitch-static sensitive otolith afferent neurones, evoked by a BCV click (acceleration shown below the unit responses)**. Reproduced with permission from Curthoys et al. (2006).

Other studies have utilized a linear BCV pulse without necessarily controlling for jerk, and most often recording the VsEP from the facial nerve canal (Böhmer, 1995; Kingma and Wit, 2009, 2010; Brown et al., 2013; Chihara et al., 2013). These later studies have all utilized simultaneous acoustic masking to suppress ECochG responses evoked by the BCV click stimulus. Importantly, click-like BCV stimulation can induce a highly synchronized response of the vestibular afferents (Figure 6; Curthoys et al., 2006), where typically only one spike is initiated by the BCV pulse, but the latency of this spike relative to the peak skull acceleration may vary slightly (by 0.2 ms to 0.5 ms) between afferent neurons. This latency variability is most likely related to the indirect nature of measuring skull acceleration as a means of interpreting the displacement of otolith HCs, although it may also demonstrate variability in the response of different HCs to a given vibration of the vestibular end-organ. Regardless of this slight variability, single-unit recordings suggest that the histogram of afferent responses to a BCV-click should be highly synchronized, and therefore the VsEP response should provide a faithful representation of the vestibular nerve field potential. This raises a question—what are the later peaks in the VsEP recorded from the facial nerve canal (Figure 4A)? Are they derived from brainstem activity, or are they a result of a complex resonance of the skull producing multiple successive VsEP responses, or are they the result of different vestibular afferent nerve responses to the BCV stimulus?

Chihara et al. (2013) attempted to determine if the later peaks were the result of a skull-resonance, evoking multiple vestibular nerve responses. Here, we (the experiments were performed in the author’s laboratory) used an audiometric bone conductor rigidly attached to the skull of a guinea pig, with an accelerometer placed nearby on the skull, to deliver a brief linear-BCV stimulus that resulted in an acceleration profile that had minimal later peaks or resonant features (Figure 4B). Acoustic masking was used to suppress cochlear responses. This approach reduced some, but not all of the later components in the VsEP response. Again, it should be realized that skull acceleration responses, particularly at high frequencies, are unlikely to represent the vibration of the end-organ. We have now abandoned this approach, and instead simply deliver brief (0.2–4 ms) monophasic pulses to the bone conductor, which is attached to the ear-bar (Brown et al., 2016). The later peaks in the VsEP responses remain, but we have so far been unable to clarify their origin.

Regardless of the exact vibration of the vestibule, using variants of this setup, several studies have demonstrated that the linear-BCV evoked VsEP is a response of otolith organs. That is, the VsEP remains after cochlear extirpation, or SCC plugging, but is abolished after death (Jones and Jones, 1999; Plotnik et al., 1999b). Moreover, selective otolith destruction abolishes the linear VsEP (Chihara et al., 2013), and otoconia deficient mice have absent or reduced VsEP responses (Jones et al., 1999, 2004). A few studies (Freeman et al., 1999a; Plotnik et al., 1999a) have attempted to stimulate selected vestibular end-organs with pulsed BCV applied in either the naso-occipital, dorso-ventral, or inter-aural directions (along with rotatory pulses), and found similar VsEP response waveforms evoked by all stimuli, but with different response amplitudes. Moreover, Jones et al. (2001) demonstrated in chickens that the initial directional polarity of the linear BCV (relative to the vestibular system), particularly for inter-aural directed stimuli, significantly alters the response waveform. It is not clear if such selective linear BCV stimulation permits a selective activation of the different vestibular end-organs, but this result highlights that that the VsEP is, at least partly, directionally sensitive.

Whilst the linear-BCV evoked VsEP is believed to originate from otolith afferent neurons, several studies have suggested that different stimuli, such as a rapid rotation of the head may generate a SCC afferent VsEP response (Elidan et al., 1982, 1987b; Li et al., 1993; Freeman et al., 1999b; Sohmer et al., 1999). Other studies have used brief low-frequency sinusoidal ACS tones, with fenestration of a given SCC canal, to stimulate a nerve response from the SCC (Wit et al., 1981; Curthoys, 2017). Some studies have suggested that high-intensity ACS can stimulate SCC afferent neurons (Zhu et al., 2014), whereas others have suggested that it does not (Curthoys et al., 2006; Curthoys, 2017). Certainly, it would seem that the otoliths are far more sensitive to transient ACS or BCV than the SCCs. Ultimately, the majority of VsEP studies that have performed additional experimental measures to investigate the origin of the VsEP response, such as selective end-organ ablation, have used a linear-BCV stimulus, and currently more evidence is required to demonstrate that a VsEP can be evoked via a stimulus designed to selectively, or preferentially activate the SCCs afferent neurons.

#### Reducing Artifacts and Cochlear Contributions

There are several potential pitfalls that need to be considered when recording EVestG responses. First, most EVestG responses are evoked using BCV stimuli generated by an electrodynamic shaker. This can produce a significant amount of electromagnetic radiation, which should be prevented from radiating to the electrodes using standard techniques such as shielded or twisted cables, and electrical and magnetic shielding of the shaker with grounded MU-metal shielding (Ford et al., 2004). Moreover, BCV of the head can produce significant electrode movement artifact, although electrode stabilization techniques can be of benefit (Comert and Hyttinen, 2015). Using alternating polarity (i.e., reverse direction) BCV stimulation can attenuate much of the artifact in VsEP measurements, but this should only be employed if the VsEP has the same waveshape and latency for either polarity stimuli, otherwise responses may partially cancel. Jones et al. (2002) demonstrated that the VsEP amplitude changed slightly with stimulus polarity, but the latency did not,^2^ and therefore alternating polarity responses could be averaged together to minimize any electrical or movement artifact, with minimal changes to the VsEP waveshape. Both Plotnik et al. (1997) and Jones et al. (2002) demonstrated that the amplitude of the VsEP decreased by up to 15% with increasing stimulus presentation rates, suggesting that an ideal rate should be around 16 per second, which is similar to the ideal repetition rate used for ECochG responses (Eggermont, 1974).

In order to suppress ECochG responses from VsEP recordings, most studies have utilized broad-band acoustic masking noise. This is often necessary because transient BCV stimuli can produce an acoustic click that is transmitted to the cochlea either as an ACS or through direct BCV (Puria and Rosowski, 2012). Acoustic masking noise can either be presented simultaneously with BCV stimulus (Böhmer, 1995; Jones and Jones, 1999; Oei et al., 2001; Chihara et al., 2013), or it can be silenced immediately prior to it (Jones T. A. et al., 2011; King et al., 2017), where forward-masking effects are sufficient to suppress any cochlear responses (Verschooten et al., 2012). It’s not clear if the primary purpose for silencing the masking noise just prior to the BCV stimulus is because the masking noise itself generates CM or electrical artifact, which can contaminate the VsEP response, or if it is believed that the acoustic masking noise may directly interfere with the BCV stimulation of the vestibular system. Several studies have suggested that high levels of noise (>110 dB SPL) can reduce the linear-VsEP amplitude (Böhmer, 1995; Sohmer et al., 1999), particularly if there is a fenestration of the SCC (Wit et al., 1981; Biron et al., 2002). This suggests that the otolith jerk-responsive HCs may be sensitive to high levels of ACS, as is known from single-unit recordings (Curthoys and Vulovic, 2011), and studies have demonstrated that loud noise exposure can produce a permanent reduction in the VsEP (Biron et al., 2002), although this conflicts with previous studies (Sohmer et al., 1999). Nevertheless, moderate continuous or forward-masking acoustic noise most likely provides an adequate suppression of cochlear activity, without overly attenuating otolith responses. Interestingly, Jones and Jones (1999) and Jones et al. (2002) suggest that VsEP responses, recorded with sub-cranial electrodes, are often unaffected by forward masking noise, suggesting that there is little contamination from ABR. This likely reflects the fact that they use a stimulus designed to maximize jerk stimulation of the otoliths, whilst minimizing cochlear stimulation.

Lastly, whilst several studies have demonstrated that the VsEP is a response of peripheral and central vestibular neurones (Nazareth and Jones, 1998; Jones and Jones, 1999; Jones et al., 2002), some studies have suggested that the VsEP measured within the inner ear can contain components that reflect vestibular *HCs* activity (Wit et al., 1986, 1990). This raises the possibility that there may be an SP-like component of the VsEP when it is measured close to the vestibular HCs. Moreover, it suggests that it may be possible to measure vestibular HCs responses, such as VM, from electrode montages that enable recording of both vestibular nerve and HCs activity.

#### Interpretation of the VsEP

A concern with interpreting VsEP responses is the uncertainty of which vestibular end-organs contribute to the response. That is, BCV stimuli can induce neural responses from all vestibular end-organs, despite primarily activating otolithic irregular afferent neurons (Curthoys et al., 2006). Whilst researchers have attempted to use the direction of the applied BCV to activate selected vestibular HCs, it is unlikely that this circumvents the complex 3-dimensional vibration of the inner ear and the complex transduction pathways (Stenfelt, 2015, 2016; Chhan et al., 2016). Mechanical engineers are well aware of the complexity of interpreting the vibrational response of a structure via its “impulse response”. An alternative method involves measuring the “steady-state” or continuous vibrational response, where the complexities of the impulse response have dissipated. For the vestibular system, this would involve measuring its response to a continuous sinusoidal linear (or rotatory) BCV stimulus, which should provide a stimulation of the vestibule that is easier to interpret, and would provide a response that could be more readily compared to single-unit recordings obtained during sinusoidal BCV (Curthoys et al., 2006; Curthoys and Vulovic, 2011). Indeed, a few studies have demonstrated that a continuous sinusoidal stimulus can evoke both a sinusoidal VM (Wit et al., 1986) and cyclic neural responses (Wit et al., 1981, 1986 Figure 7). These responses are reminiscent of the auditory nerve neurophonic, used to assess low-frequency sensitivity of the cochlea during a tone (Henry, 1995; Lichtenhan et al., 2014). It may therefore be possible to use sinusoidal ACS or BCV to evoke vestibular neurophonic, and this may provide a means to obtain responses from vestibular neurones which are most sensitive to vibration in a specific direction. Meanwhile, the VsEP obtained using impulse stimuli should assume that the VsEP is “mostly” a response of the afferent neurons synapsing with the jerk-sensitive HCs in the otoliths, with some potential contributions from all vestibular end-organs (see “VsEP Stimulus” Section).

**Figure 7:**
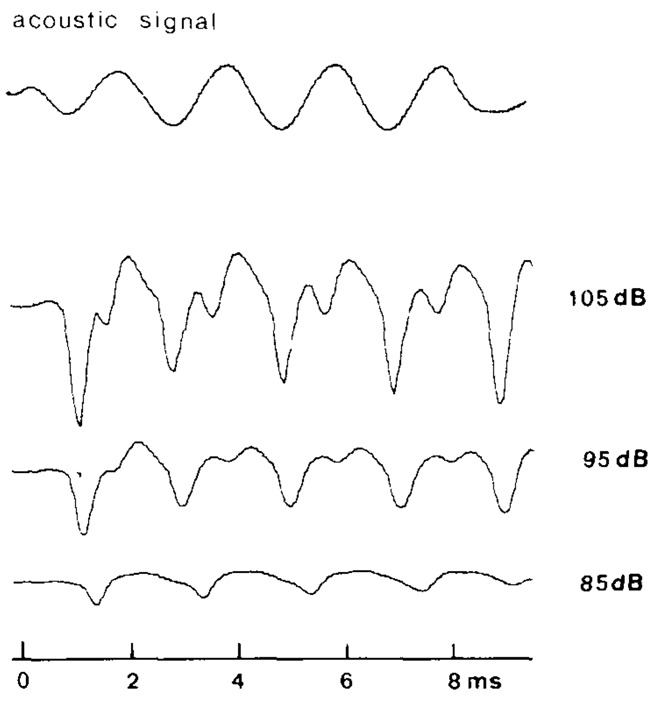
**Vestibular nerve responses, measured from a scala tympani electrode in pigeons, after cochlear extirpation and SCC fenestration, evoked by a 500 Hz ACS toneburst (upper trace) of sound levels between 85 dB SPL and 105 dB SPL**. It is thought that these responses reflect a neurophonic of the vestibular nerve. Reproduced with permission from Wit et al. (1981).

Whilst it may be tempting to use static tilts to probe the origin of the VsEP response, the issue of static head position during VsEP measurements is one which still needs to be resolved. Plotnik et al. (1999a) suggested that, in addtion to changes related to stimulus delivery direction, VsEP responses were altered by the static orientation of the head, suggesting that gravity may alter the sensitivity of the jerk-sensitive HCs. This contrasts with a lack of static head-orientation changes in similar measures otolith function in humans (Kastanioudakis et al., 2016).

Encouragingly, for researchers using the VsEP as a measure of peripheral vestibular function in longitudinal studies, Honaker et al. (2015) demonstrated that the VsEP amplitude and threshold do not change significantly across repeated recordings, which includes repositioning of electrodes (at fixed/standardized positions). Thus, as long as the delivery of the BCV stimulus is consistent between successive recording sessions, the VsEP should provide a sensitive measure of changes in peripheral vestibular sensitivity. It should be noted that response variability will also depend on the signal-to-noise ratio of the response, which greatly depends on the number of averages. For the VsEP measured at the vertex, the response is typically averaged of over 200 times, due to the low signal-to-noise ratio (Jones et al., 2002). To reduce variability in the responses due to the noise-floor of the recording, responses can be band-pass filtered between 300 kHz and 10 kHz (Jones et al., 2002), although these filter settings were obtained for VsEP responses recorded at the vertex, and may differ for VsEP responses measured in the periphery.

An important factor to consider when monitoring VsEP responses during an intervention, is how to assess changes. Previously, many studies have monitored the peak-to-peak amplitude of the response, however because the later peaks in the VsEP reflect central responses, they may be altered without an equivalent change in the 8th nerve’s sensitivity, resulting in changes in the VsEP waveform (Jones et al., 2000; Morley et al., 2017). Therefore, VsEP thresholds should ideally be used to assess changes in the sensitivity of the irregular otolith afferents, although changes in the VsEP waveform, such as changes in inter peak intervals and peak latencies, may provide additional information. That said, the source of the later peaks in VsEP responses recorded from the vertex is not as well defined as the origin in the later peaks in ABR responses (Kaga et al., 1997), although several studies have used electrical source analysis to localize VsEP activity (Todd et al., 2014, 2017).

One final issue to consider is the potential influence of anesthetics on EVestG responses (Gaines and Jones, 2013). Although anesthesia is known to suppress certain cortical activity, there seems to be little difference in the VsEP measured at the vertex, between anesthetized and awake animals, other than a suppression of a late (>7 ms) component, which may potentially reflect cortical vestibular activity (Jones, 1992). Nonetheless, it is possible that different anesthetics may induce changes in the VsEP response, particularly of the later, central components.

## Human EVestG Recordings

Other than the recent controversial asynchronous-EVestG responses recorded on the tympanum in humans (Lithgow, 2006; Lithgow et al., 2008; Dastgheib et al., 2016), several studies have reported on VsEP responses measured in humans, with virtually no human VM recordings. Elidan et al. (1991b), and Rodionov et al. (1996) recorded small (0.5 μV peak to peak) short latency potentials from the forehead (with a mastoid inverting electrode), in response to rapid angular rotations of the head (10,000°/s^2^). Similarly, Pyykkö et al. (1995) measured small VsEP responses evoked by brief linear BCV stimulation in people. Both short-latency (starting 2 ms to 3 ms) and larger middle-latency (starting 8 ms to 10 ms) responses were observed in these studies, and it was suggested that the first positive peak of the short-latency responses reflected activity of the peripheral vestibular nerve. The responses were not present in cadaver heads, or subjects with bilateral vestibular loss, but they were present in deaf subjects. These rotationally evoked human responses were compared to the VsEP responses measured in cats using a similar stimulus and measurement protocol (Li et al., 1993), and were believed to reflect responses of the SCC afferents and central vestibular neurons. Knox et al. (1993) recorded similar short latency vestibular responses to rapid whole-body linear accelerations, measured between the forehead and mastoid, and suggested the early components of their responses reflected the activity of the peripheral vestibular nerve from otolith neurons. Ultimately, each of these human VsEP displayed a poor signal-to-noise ratio, and required an elaborate setup to produce controlled acceleration of the head, which induced significant artifact.

de Waele et al. (2001) electrically stimulated the 8th nerve in 11 patients undergoing vestibular nerve section for Meniere’s disease, and recorded evoked responses occurring 3–5 ms after stimulation, with 30 subcutaneous electrodes placed on the scalp. Electrical source analysis was used to localize the response activity to various regions of the brain, including an early component localized to the region of the vestibular nucleus. This study supported the theory that vestibular information is processed in spatially distributed central pathways, rather than at a focal cortical region (Cullen, 2016). It should be noted that de Waele et al. (2001) suggested their electrically evoked response reflected the activity of central vestibular neurones only, and that the activity of the peripheral vestibular system, including the 8th nerve, was not represented in the response.

More recently, several studies have suggested that vestibular responses, termed VsEPs, to loud (>100 dB SPL), low frequency (e.g., 500 Hz) acoustic tone bursts can be recorded with electrodes placed at the vertex (Todd et al., 2003, 2014; McNerney et al., 2011). Certainly it has been shown that the human vestibular system, particularly the otoliths, is sensitive to acoustic tones (Chihara et al., 2009; Murofushi et al., 2010). Moreover, the origin of these short latency scalp potentials were localized to various brain regions known to be related to central vestibular pathways (Todd et al., 2003, 2014). However, like the responses reported by de Waele et al. (2001), no components were localized to the peripheral vestibular system, such as the 8th nerve. Here, it appears that human scalp VsEP responses are similar to the later components observed in experimental animal VsEPs (Nazareth and Jones, 1998). Moreover, recent human scalp VsEP recordings have demonstrated that the amplitude of components of this response can be modulated by head and eye position (Todd et al., 2017), which reflects their central origin. Thus, caution should be taken when using human VsEP responses as an estimate of peripheral vestibular function, because like vestibular reflex responses, central vestibular activity may not faithfully reflect the sensitivity of the peripheral vestibular system.

Here we ask the question, what is the advantage of EVestG as a measure of vestibular sensitivity compared to several reflex measures of vestibular function clinically (Curthoys, 2012; Colebatch et al., 2016). For experimental animal researchers the answer is clear—it can be difficult, but not impossible, to measure vestibular reflexes in anesthetized animals because central reflex pathways and myogenic activity is heavily suppressed (Vulovic and Curthoys, 2011). Experimental animal research has traditionally relied on objective measures of vestibular activity, such as single-unit recordings or gross HCs and nerve responses. However, the modulation of vestibular reflexes highlights an additional need to develop objective measures of peripheral vestibular function in humans. These responses, whilst typically robust and incorporating only three or four neurons in the reflex pathway, can adapt and may be modulated by central mechanisms (Mantokoudis et al., 2016). Thus, the clinical diagnosis of vestibular disorders would likely benefit from measures of peripheral vestibular function, similar to how ECochG has been used in the diagnosis of several inner ear disorders, such as Meniere’s disease, 8th nerve schwannomas, auditory neuropathy, and sudden sensorineural hearing loss (see Eggermont, 2017).

## Utility of EVestG in Research

Increasingly, the linear BCV evoked VsEP is being used in experimental animals to improve our understanding of both fundamental and pathological peripheral vestibular function. The VsEP has been studied in animal models of otoconia deficiencies (Jones et al., 1999, 2004; Zhao et al., 2008), aging (Mock et al., 2011; Vijayakumar et al., 2015), hyper-gravity (Jones et al., 2000), gentamicin treatment (Perez et al., 2000; Bremer et al., 2014; King et al., 2017), endolymphatic hydrops (Kingma and Wit, 2009, 2010; Chihara et al., 2013), diuretic effects (Bremer et al., 2012), anesthetics (Gaines and Jones, 2013), pharmacological agents (Irons-Brown and Jones, 2004), inner ear genetic disorders (Jones S. M. et al., 2011; Lee et al., 2013; Robertson et al., 2008; Mathur et al., 2015), and noise trauma (Sohmer et al., 1999; Biron et al., 2002). More recently, studies have demonstrated abnormal VsEP responses in knockout mice lacking nicotinic acetylcholine receptors (Morley et al., 2017), which are expressed at the peripheral vestibular efferent synapse (Holt et al., 2015), on vestibular HCs (Simmons and Morley, 2011), and within peripheral and central vestibular neurons (Happe and Morley, 1998). Additionally, there is an increasing interest in utilizing EVestG as a means to uncover the functional role of the vestibular efferent system, in much the same way the cochlear CAP and CM have been used to study the functional role of the olivocochlear efferent neurones (Gifford and Guinan, 1987; Elgueda et al., 2011; Lichtenhan et al., 2016).

Importantly, it should be recognized that the VsEP provides only a limited measure of peripheral vestibular function. That is, research suggests that the BCV evoked VsEP is primarily a response of the neurons innervating jerk-sensitive HCs on the otoliths. The corollary of this is that the VsEP does *not* provide a measure of neurones innervating static-sensitive HCs, such as those in the extra-striola regions, or the SCCs, and moreover it does not provide a measure of HCs function. Therefore, the VsEP should not be used as a measure of overall vestibular sensitivity. Experimental manipulations or pathologies that alter the function of extra-striola or SCC HCs, are unlikely to produce significant changes in the VsEP. There are several pathologies that affect SCC but not otolith function (e.g., Meniere’s disease; McGarvie et al., 2015), or affect the superior nerve (which innervates the SCC and most of the utricle; Curthoys et al., 2009), but not inferior nerve (e.g., superior vestibular neuritis; Curthoys et al., 2011). Moreover, the VsEP is a neural response, and should not be used as a definitive indicator of vestibular HCs function. Auditory neuropathy spectrum disorder is an example pathology of a pathology which affects peripheral nerve but not HCs function (Stuermer et al., 2015; Kim et al., 2016). Lastly, precisely which HCs and neurones are responsible for generating the VsEP is still not entirely clear. That is, whilst evidence points towards the VsEP being a response of jerk-sensitive HCs/neurons, this may need further clarification, particularly given that different forms of BCV stimulation, in different experimental animals, may stimulate various the sub-sets of the peripheral vestibular system.

As studies continue to demonstrate changes in the VsEP due to genetic abnormalities or pharmacological treatments, with little or no change in tissue morphology (Lee et al., 2013; King et al., 2017; Morley et al., 2017), there may be a need to differentiate the cause of the functional loss as either HCs or neural dysfunction, and it is here that VM may be employed. When recorded from the inner ear fluids, the VM is a “global” response from all vestibular HCs types, because all vestibular HCs respond to low-frequency stimulation, and the extracellular potentials will summate in the fluids. Such a global VM measure is of limited use as a measure of peripheral vestibular function. However, it may be possible to obtain a “local” VM measure from specific HCs, if the VM is recorded with glass micropipettes localized in close proximity to the HCs ( Pastras et al., under review). Currently, there is a need to further develop techniques for measuring vestibular HCs receptor potentials or currents *in vivo*.

Lastly, there are few studies monitoring evoked EVestG responses in humans. One area in which both ECochG and EVestG are rapidly developing is as an intraoperative monitor of inner ear function during inner ear surgeries such as the insertion of cochlear and vestibular implants (Frijns et al., 2002; Campbell et al., 2015, 2016; Scott et al., 2016). Like the electrically evoked CAP (eCAP) component of “neural response telemetry”, the electrically evoked VsEP (vestibular eCAP, or eVsEP) represents the electrically evoked response of the vestibular nerve (Nie et al., 2011). As the vestibular implant continues to be developed for chronic vestibular disorders, the eVsEP is likely to play an important role in the surgical positioning of the implant electrodes within the vestibular system, and objectively assessing the implants efficacy over time, as a supplement to monitoring the electrically evoked vestibular reflex responses when patients are awake.

## Conclusion

Foremost, EVestG presents a simple tool to monitor vestibular function in animal experiments. Currently, VsEPs are the most prevalent EVestG responses measured in experimental research, and the test setup and protocol developed by Jones and Jones (1999), for use in mice and rats, largely dominate the field. Gradually more research laboratories, such as ours, are incorporating VsEP measurements, and experience suggests that it is vital to have a clear understanding of the potential pitfalls of EVestG measurements. That is not to suggest new EVestG techniques cannot be developed to suit individual research needs, and certainly we anticipate that EVestG measurement techniques will evolve much the same way new ECochG techniques are being developed. Particularly, techniques for measuring both the VM and the VsEP simultaneously (Wit et al., 1981, 1986), as in the case of the cochlear CAP and CM, are likely to help address several key “unknowns” in vestibular research, such as the role the vestibular efferents play (Morley et al., 2017).

Human EVestG responses haven’t shown much promise to date; either because they are exceptionally small compared to the noise floor, or because they have been entirely superseded by a host of vestibular reflex tests that permits a rapid assessment of the peripheral vestibular system, with minimal central processing. It’s unlikely that EVestG could be monitored from the tympanum or round-window, as is the case with ECochG, but certainly as the vestibular implant continues to develop, researchers may be able to leverage the proximity of the electrodes to the vestibular nerve to obtain clear vestibular responses in humans.

Finally, just as there are a host of terms given to differential ECochG measures, new terminology should be developed for EVestG responses, either drawing on comparative terms that have been applied to cochlear responses, or being based more on the logical appreciation of what the response represents. However, given the overlap between cochlear and vestibular research, it would seem more appropriate to utilize terminology that has already been developed for cochlear responses.

## Author Contributions

DJB developed the review and wrote the manuscript. ISC and CJP edited the manuscript, and provided additional input to the content.

## Conflict of Interest Statement

The authors declare that the research was conducted in the absence of any commercial or financial relationships that could be construed as a potential conflict of interest.

## Footnotes

^1^No attempt has been made to perform a validated systematic review, but the large discrepancy in the numbers do not warrant such an approach.

^2^It should be noted that Jones et al. (2002) were able to push and pull the skull, and that under different stimulus conditions, there may be a difference in the latency of the VsEP due to a difference in the BCV transduction.
